# First experience with real-time magnetic resonance imaging-based investigation of respiratory influence on cardiac function in pediatric congenital heart disease patients with chronic right ventricular volume overload

**DOI:** 10.1007/s00247-023-05765-9

**Published:** 2023-10-05

**Authors:** Lena Maria Röwer, Karl Ludger Radke, Janina Benner, Halima Malik, Monika Eichinger, Dirk Voit, Mark Oliver Wielpütz, Jens Frahm, Dirk Klee, Frank Pillekamp

**Affiliations:** 1 Department of Diagnostic and Interventional Radiology, Medical Faculty and University Hospital Düsseldorf, Heinrich-Heine-University, Moorenstr. 5, 40225, Düsseldorf, Germany; 2 Department of General Pediatrics, Neonatology and Pediatric Cardiology, Medical Faculty and University Hospital, Heinrich-Heine-University, Düsseldorf, Germany; 3 Department of Diagnostic and Interventional Radiology with Nuclear Medicine, Thoraxklinik at University of Heidelberg, Heidelberg, Germany; 4 Department of Diagnostic and Interventional Radiology, Subdivision of Pulmonary Imaging, University Hospital of Heidelberg, Heidelberg, Germany; 5 Translational Lung Research Center Heidelberg (TLRC), German Center for Lung Research (DZL), Heidelberg, Germany; 6 Biomedical NMR, Max Planck Institute for Multidisciplinary Sciences, Göttingen, Germany; 7 DZHK (German Centre for Cardiovascular Research), Partner Site Göttingen, Göttingen, Germany

**Keywords:** Cardiac magnetic resonance imaging, Congenital heart disease, Heart–lung interaction, Pediatric, Real-time imaging

## Abstract

**Background:**

Congenital heart disease (CHD) is often associated with chronic right ventricular (RV) volume overload. Real-time magnetic resonance imaging (MRI) enables the analysis of cardiac function during free breathing.

**Objective:**

To evaluate the influence of respiration in pediatric patients with CHD and chronic RV volume overload.

**Methods and materials:**

RV volume overload patients (*n*=6) and controls (*n*=6) were recruited for cardiac real-time MRI at 1.5 tesla during free breathing. Breathing curves from regions of interest reflecting the position of the diaphragm served for binning images in four different tidal volume classes, each in inspiration and expiration. Tidal volumes were estimated from these curves by data previously obtained by magnetic resonance-compatible spirometry. Ventricular volumes indexed to body surface area and Frank-Starling relationships referenced to the typical tidal volume indexed to body height (TTVi) were compared.

**Results:**

Indexed RV end-diastolic volume (RV-EDVi) and indexed RV stroke volume (RV-SVi) increased during inspiration (RV-EDVi/TTVi: RV load: + 16 ± 4%; controls: + 22 ± 13%; RV-SVi/TTVi: RV load: + 21 ± 6%; controls: + 35 ± 17%; non-significant for comparison). The increase in RV ejection fraction during inspiration was significantly lower in RV load patients (RV load: + 1.1 ± 2.2%; controls: + 6.1 ± 1.5%; *P*=0.01). The Frank-Starling relationship of the RV provided a significantly reduced slope estimate in RV load patients (inspiration: RV load: 0.75 ± 0.11; controls: 0.92 ± 0.02; *P*=0.02).

**Conclusion:**

In pediatric patients with CHD and chronic RV volume overload, cardiac real-time MRI during free breathing in combination with respiratory-based binning indicates an impaired Frank-Starling relationship of the RV.

**Graphical Abstract:**

**Figure:**
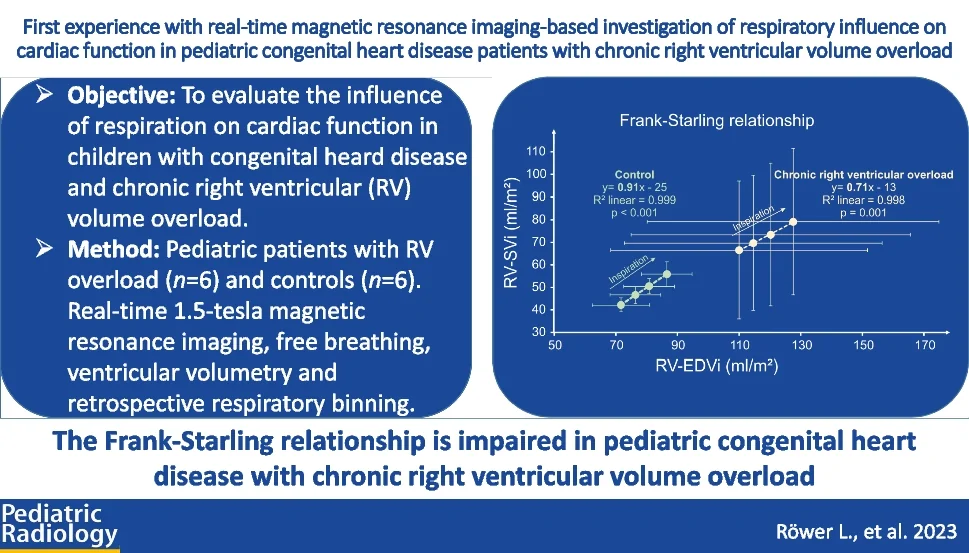

**Supplementary Information:**

Supplementary material is available at https://doi.org/10.1007/s00247-023-05765-9.

## Introduction

Congenital heart disease (CHD) is frequently associated with chronic volume overload of the right ventricle (RV) causing right heart failure in the long term (e.g., pulmonary regurgitation after surgery for tetralogy of Fallot, dilatation of congenital pulmonary valve stenosis, or left-to-right shunt in atrial septal defects) [1–4]. Although chronic RV volume overload is usually well tolerated over many years and impressive remodeling can be expected especially after closure of atrial septal defects [5, 6] and in patients with tetralogy of Fallot [7–9], right heart dysfunction has been proven to be a relevant determinant of long-term outcome in pediatric and adult patients with CHD [10]. Pre- and afterload of the RV are substantially modified by breathing [11, 12]. Therefore, the influence of respiration on function and dimensions (e.g., by modifying the preload) might be a relevant parameter in the assessment of cardiac function.

Compared to conventional cardiac magnetic resonance imaging (MRI), which requires breath-holding and only provides a low temporal resolution, real-time MRI enables the analysis of cardiac function under preserved physiological conditions during free breathing with high sampling rates [13–18]. Therefore, cardiac real-time MRI is highly beneficial for use in children, who often have difficulties with breath-holding and have higher heart and respiratory rates in comparison to adults.

In addition, the combination of cardiac real-time volumetry during free breathing with respiratory-based binning using magnetic resonance-compatible spirometry provides a unique opportunity to assess the dynamics of heart–lung interactions [14], even allowing the non-invasive investigation of the Frank-Starling relationship as a pivotal mechanism of ventricular function. The Frank-Starling relationship states that an increase in preload (e.g., due to increased venous inflow) increases the length of cardiac fibers. Therefore, a higher ventricular pressure can be produced, resulting in a higher stroke volume [14, 19–21].

Breath-holding is a nonphysiological and static condition precluding the analysis of the dynamics of respiratory modification of cardiac function. In contrast, real-time MRI during free breathing provides a physiological situation in which to study the dynamics of heart–lung interactions. Such dynamic measurements have already been shown to be superior to static parameters in other scenarios, e.g., in a mini-fluid challenge for assessing fluid responsiveness [22].

Currently, therapy-related parameters and estimation of prognosis are based on the analysis of cardiac function and dimensions acquired with conventional cardiac MRI under nonphysiological conditions. For example, in pediatric patients with tetralogy of Fallot, preoperative thresholds of RV volumes for surgical pulmonary valve replacement rely on conventional cardiac MRI during breath-holding [7–9, 23–28].

To date, no study has examined cardiac function in pediatric patients with RV volume overload under physiological conditions using cardiac MRI. In particular, the Frank-Starling mechanism has not yet been studied in children with chronic RV volume overload.

The aim of this study was to investigate the respiratory influence on cardiac function in pediatric patients with CHD and chronic RV volume overload. We hypothesized that the respiratory-induced alterations of preload and the adaptability of the heart to further volume load, i.e. chronic RV volume overload, modifies the Frank-Starling mechanism. We further hypothesized that alteration of the Frank-Starling mechanism in patients with RV volume overload can become an additional meaningful diagnostic parameter to assess ventricular function and to add to the information provided by static parameters.

## Material and methods

### Experimental design

The study is a descriptive, retrospective real-time MRI study of pediatric patients at a tertiary children’s hospital (Düsseldorf University Hospital). The parents and/or their legal guardians signed a written declaration of consent. The study was approved by the local ethics committee (Ethics Committee of the Medical Faculty, Düsseldorf University Hospital, study number 6176R).

A total of six pediatric patients (Table 1) with significant RV volume overload caused by congenital heart disease and six pediatric controls without volume overload and with a normal cardiac MRI examination were included in the study. Controls had no underlying cardiovascular or pulmonary disorders influencing the filling pressure. Significant RV volume overload was defined as an RV end-diastolic volume indexed to body surface area (RV-EDVi) determined with conventional cardiac MRI volumetry in end-expiration above the age-related 90th percentile [29]. DuBois' formula (body surface area [m^2^]=weight [kg]^0.425^ × height [cm]^0.725^ × 0.007184) was used to calculate the body surface area for indexed data.

**Table 1:** Patient characteristics and indications for cardiac magnetic resonance imaging (MRI)

		Patient information								Indication for cardiac MRI	
Patient		Age (years)	Sex	Body weight (kg)	Body height (cm)	Body surface area (m^2^)	Heart rate (bpm)	Respiratory rate (breaths/min)	RV-EDVi (ml/m^2^)	Diagnosis	Indication for MRI
Chronic RV overload	1	7	Female	24	130	0.93	100	26	199	Sinus venosus atrial septal defect	RV function and dimension, Qp/Qs
	2	10	Male	27	130	0.99	86	18	122	Tetralogy of Fallot, pulmonary regurgitation and moderate stenosis, peak RVOT gradient 33 mmHg	RV function and dimension
	3	9	Male	29	133	1.04	84	24	144	Tetralogy of Fallot, pulmonary regurgitation and moderate stenosis (peak RVOT gradient 25 mmHg)	RV function and dimension
	4	5	Female	20	116	0.81	77	25	88	Tetralogy of Fallot, pulmonary regurgitation and moderate stenosis (peak RVOT gradient 41 mmHg)	RV function and dimension
	5	9	Male	32	135	1.10	83	16	88	Partial anomalous venous drainage	RV function and dimension
	6	12	Female	46	155	1.42	84	18	87	Tetralogy of Fallot, pulmonary regurgitation and mild stenosis (peak RVOT gradient 17 mmHg)	RV function and dimension
Controls	7	13	Female	43	153	1.35	83	17	67	-	Exclusion of atrial septal defect
	8	16	Male	48	165	1.48	109	20	84	-	Exclusion of atrial septal defect
	9	17	Female	67	163	1.74	92	17	80	-	Exclusion of atrial septal defect
	10	15	Male	58	181	1.75	75	18	78	-	Exclusion of atrial septal defect
	11	15	Female	78	172	1.91	60	19	71	-	Exclusion of myocarditis
	12	13	Male	40	156	1.34	83	12	82	-	Exclusion of myocarditis
Chronic RV overload mean ± SD		9 ± 2	Male=3 Female=3	30 ± 8	133 ± 12	1.0 ± 0.2	86 ± 7	21 ± 4	123 ± 40	-	-
Controls mean ± SD		15 ± 2	Male=3 Female=3	56 ± 14	165 ± 9	1.60 ± 0.22	84 ± 15	17 ± 3	77 ± 6	-	-
Comparison		*P*<0.01	-	*P*<0.01	*P*<0.01	*P*<0.01	*P*>0.05	*P*=0.04	*P*=0.03		

*bpm* beats per minute, *LV* left ventricle/ventricular, *Qp* pulmonary blood flow, *Qs* systemic blood flow, *RV* right ventricle/ventricular, *RV-EDVi* right ventricular end-diastolic volume indexed to body surface area, *RVOT* right ventricular outflow tract, *SD* standard deviation

### Imaging protocol

The cardiac MRI measurements were performed on a clinical 1.5-tesla MRI scanner (MAGNETOM Avanto fit, Siemens Healthineers, Erlangen, Germany; software version Syngo MR E11) with an installed 32-channel spine matrix coil (direct connect spine 32) and an 18-channel body coil (Body 18, both Siemens Healthineers) in supine position. The MRI protocol began with a standard clinical pediatric CHD protocol including conventional cardiac localizers and retrospectively gated four-chamber and two-chamber cine datasets acquired in end-expiratory breath-holding for planning the short axis stack, phase-contrast imaging of the pulmonary and aortic flow, and additional sequences if clinically indicated [30]. A real-time MRI sequence acquired a series of 900 cross-sectional real-time magnetic resonance images in each slice of the short axis stack, covering the left and right ventricles during free breathing. For detailed sequence information, see Table 2. The slice orientation of the short axis stack was perpendicular to the interventricular septum. Slice thickness was 8 mm without a gap between slices (distance factor 0%) irrespective of age, in order to maintain consistency with our research protocol. The duration of the real-time MRI examination varied depending on the number of ventricular slices to cover the heart (RV overload: 11 slices [in four patients], 12 slices [in two patients]; controls: 10 slices [in one control], 11 slices [in two controls], 12 slices [in three controls]) and lasted 30 s for each slice. During the real-time MRI data acquisition, Siemens Signal logging VD11a ECG UNIT, PERU 098 Siemens Healthineers) was used to record the electrocardiograms (ECG).

**Table 2:** Sequence parameters for real-time magnetic resonance imaging (MRI)

Sequence parameters	Real-time MRI
Sequence type	2D b-SSFP
TR/TE (ms)	3.7/1.85
FOV (mm)	316–500
Image matrix (pixels)	200
Inplane resolution (mm × mm)	1.6 × 1.6
Slice thickness (mm)	8
Interslice gap (mm)	0
Phases	900
Orientation	Short axis
Flip angle (°)	60
Bandwidth (Hz/pixel)	760
Image acquisition time (ms)	33

*b-SSFP* balanced steady-state free precession, *ECG* electrocardiography, *FOV* field of view, *TE* echo time, *TR* repetition time

### Binning

**Fig. 1:**
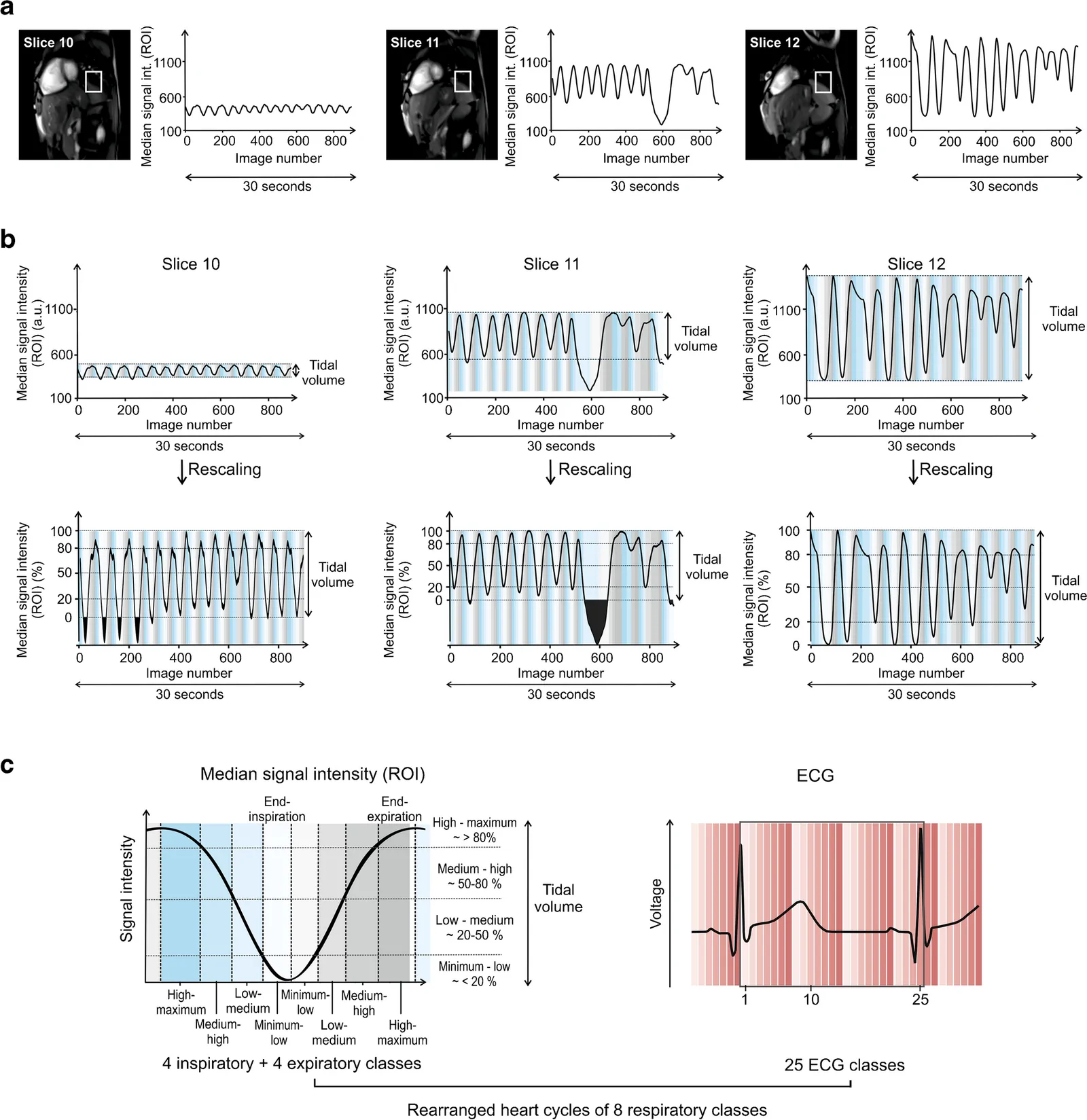
Respiratory signal*.* Exemplary illustration (slices 10–12) demonstrating the processing of image-based respiratory signals. **a** Respiratory-related diaphragmatic movement was monitored by the median SI of manually selected ROI (*white squares*). **b** Rescaling, removal of outliers, and assessment of at least five consecutive respiratory cycles resulted in a good estimate of the tidal volume. **c** Real-time magnetic resonance images were binned based on SI values and ECG in rearranged heart cycles with 25 phases in eight respiratory classes (four different tidal volume classes both in inspiration and expiration). *ECG* electrocardiogram, *MRI* magnetic resonance imaging, *ROI* region(s) of interest, *SI* signal intensity

Real-time MRIs were binned based on respiration and ECG-derived RR intervals. Information on respiration was derived from signal intensity (SI) changes related to diaphragmatic movement in regions of interest (ROIs). ROIs were positioned using the free-hand contouring tool in the series viewer module of the commercial evaluation software cvi42 (Release 5.10.1.(1241); Circle Cardiovascular Imaging Inc. Calgary, Canada) (Fig. 1).

Further real-time data processing was done in Python (v3.8.4. Python Software Foundation, Wilmington, DE), by adapting published open-source packages (e.g., Numpy [31] and pydicom [32]). For details, see https://github.com/MPR-UKD/RT-MRI-Respiratory-Cardiac-Function-CHD. The moving median based on the previous and subsequent ten SI values was used to provide the information on the respiratory phase (inspiration was defined as first derivative ≤ 0, expiration was defined as first derivative ˃ 0). The moving median values and the value of the first derivative thereof were assigned to the individual MRIs. Information on the time after the R wave of the ECG was included in the dicom tags provided by the intrinsic magnetic resonance scanner software (Syngo MR E11, Siemens Healthineers, Erlangen, Germany). SI curves were individually rescaled to eliminate respiratory outliers reflecting functional residual capacity or inspiratory reserve volume, resulting in at least five respiratory cycles for each slice within the typical tidal volume range (Fig. 1). Real-time images were then binned in eight respiratory classes (four different tidal volume classes for inspiration and expiration, respectively) and 25 cardiac phases. As previously demonstrated, this number of classes provides sufficient coverage of the respiratory classes within a reasonable scanning time [14] (Fig. 1).

In case of overfilled bins, images were filtered. For this purpose, an efficient state-of-the-art subpixel phase correlation analysis was used to compare the images [33]. Based on the mean phase difference and standard deviation (SD), images that differed significantly (deviation greater than SD) from the others were excluded. The image with SI value closest to the respective median of the respiratory volume class was selected for further analysis. In cases where a bin remained empty, it was filled with an image of a neighboring respiratory class for technical reasons but was excluded from further analysis thereafter.

### Tidal volume estimation

The relationship between the position of the heart and tidal volume at end-diastole measured in healthy, adult volunteers using MR-compatible spirometry was used to calculate estimates of the tidal volume of the respiratory classes (for details, see Supplementary Material 1 and Supplementary Material 2).

### Volumetry and Frank-Starling relationship

Ventricular volumetry was performed after respiratory and ECG-based binning for short axis stacks from eight respiratory classes that could be analyzed in the same way as conventional cardiac cine MRI stacks typically obtained during breath-hold only at end-expiration [14]. For this purpose, the Short Axis 3-dimensionalModule (cvi42 Release 5.10.1.(1241); Circle Cardiovascular Imaging Inc., Calgary, Canada) was used for automatic contouring of the left ventricular (LV) endocardial and epicardial contours and the RV endocardial contours at end-diastole and end-systole; manual corrections were performed by L.R. (MD, research assistant with three years of experience in cardiac MRI) based on a standardized approach [14] and considering current recommendations on cardiac image analysis [29, 34–36].

The Frank-Starling relationship was calculated as the ratio of the increase in the stroke volume (ΔLV-SVi, ΔRV-SVi), and the increase in the end-diastolic volume (ΔLV-EDVi, ΔRV-EDVi).

### Statistical analysis

All statistical analyses were performed in SPSS (IBM Corp. Released 2017, IBM SPSS Statistics for Windows, Version 26.0., Armonk, NY). The statistics for calibration curves were calculated using linear or quadratic regression as appropriate.

Linear regression was performed to evaluate the correlation between estimated tidal volumes and the corresponding RV and LV volumetry results and to investigate Frank-Starling relationships.

Finally, the residuals of all regression analyses were tested for normal distribution using the Kolmogorov–Smirnov and Shapiro–Wilk tests. Normally distributed data, the Frank-Starling slopes and respiratory-related ventricular volumes from RV overload patients and controls were compared using the *t*-test for independent samples.

A level of *P*<0.05 was considered significant.

## Results

### Patient data

On average, patients with chronic RV overload were significantly younger, smaller, and lighter than the controls (*P*<0.01) (Table 1). The heart rate was similar (*P*>0.05); the respiratory rate was higher (*P* = 0.04) in patients with chronic RV overload compared to controls (Table 1).

### Binning

Images were divided into 13,600 bins (RV overload: 4 × 11 slices, 2 × 12 slices resulting in 68 slices × 25 ECG classes × 8 respiratory classes=13,600; controls: 1 × 10 slices, 2 × 11 slices 3 × 12 slices resulting in 68 ventricular slices × 25 ECG classes × 8 respiratory classes=13,600). The interval between the ECG phases was similar in both groups (RV overload: 28.2 ± 2.2 ms; controls: 29.7 ± 5.6 ms, *P*=0.3). Respiratory binning resulted in a high percentage of filled bins (RV overload: 12,305/13,600 bins [90.5%]; controls: 12,197/13,600 bins [89.7%]; *P*=0.6 for comparison). The results are largely based on end-diastolic and end-systolic phases. The number of unfilled bins in these phases was similar in end-diastole (RV overload: 73/1,296 [5.6%]; controls: 62/1,403, [4.4%]; *P*=0.6) and end-systole (RV overlaod: 36/1,296 [2.8%]; controls: 60/1,403 [4.3%]; *P*=0.07).

### Tidal volume estimation

A separate calibration study demonstrated a linear relationship between cardiac movement and tidal volume indexed to height during normal breathing that was used to normalize results to allow comparison between subjects. The mean maximum tidal volume during normal breathing was 2.7 ± 0.7 ml/cm body length, 408 ± 117 ml absolute lung volume, and thus was within the range of quiet breathing. Consequently, the linear relationship was used, which best reflects tidal volumes during calm breathing assumed from the respiratory-modified cardiac position (for details, see Supplementary Material 1).

### Respiration-dependency of ventricular volumes

Respiration modified the shape and volume of both ventricles. To aid qualitative visual assessment of the effect of respiration on the heart, images of the same midventricular slice of one control subject (control no. 7 (random selection) in Table 1) that had all been acquired during the same ECG-defined phase but at different time points during the breathing cycle were saved as a movie (see Supplementary Material 3) and in an RV overload patient (RV overload patient no. 1 (patient with the highest RV load) in Table 1) (see Supplementary Material 4).

### Right ventricle

#### Qualitative analysis

Visual inspection of the midventricular slice readily demonstrated the influence of respiration on ventricular dimensions. At end-diastole, the almost triangular shape of the RV became more convex and rounded with increasing tidal volume, especially during inspiration. The respiratory-related alterations of RV dimensions were more prominent in control no. 7 in Table 1 (Supplementary Material 3) than in an RV overload patient (RV overload patient no. 1 in Table 1, Supplementary Material 4).

#### Quantitative analysis

**Fig. 2:**
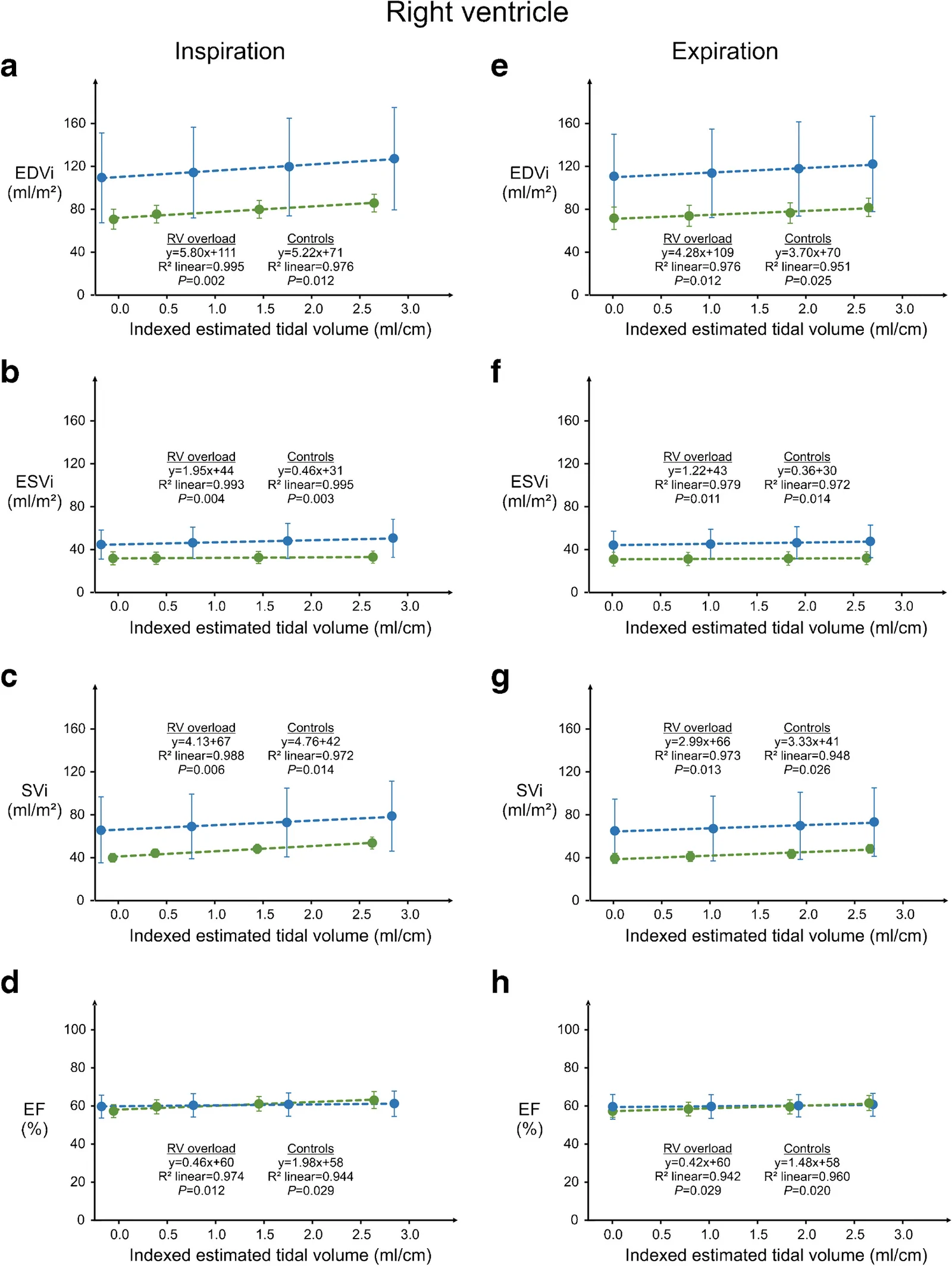
Right ventricular volumes. Mean values (± SD) of right ventricular end-diastolic volume, end-systolic volume, stroke volume, and ejection fraction indexed to the body surface area as a function of the estimated tidal volume indexed to body height in inspiration (**a**–**d**) and expiration (**e**–**h**) for patients with right ventricular overload (*blue*) and controls (*green*). Results of the corresponding linear regression analyses are inserted in the graphs*.* Circles indicate mean value, *whiskers*, SD. *P<0.05 (linear regression). *EDVi* end-diastolic volume indexed to body surface area, *ESVi* end-systolic volume indexed to body surface area, *SVi* stroke volume indexed to body surface area, *EF* ejection fraction, *SD* standard deviation

RV-EDVi, RV-SVi, and RV ejection fraction (RV-EF) per TTVi increased with increasing tidal volume both in RV overload patients and in controls, especially during inspiration (Fig. 2, Table 3).

**Table 3:** Absolute and relative changes of indexed ventricular volumes and changes of ejection fractions during inspiration

		Volume increase (percentage relative to end-expiratory volume), absolute increase of ejection fraction		*T*-test for comparison (*P*-value)
		RV overload	Controls	
RV	EDVi (ml/m^2^) per TTVi (ml/m^2^)	+ 19.0 ± 6.0 (+ 16.0 ± 9.0%)	+ 16.0 ± 7.0 (+ 22.0 ± 13.0%)	0.31
	ESVi (ml/m^2^) per TTVi (ml/m^2^)	+ 6.8 ± 4.6 (+ 1.4 ± 0.9%)	+ 12.0 ± 7.0 (+ 4.7 ± 4.3%)	0.09
	SVi (ml/m^2^) per TTVi (ml/m^2^)	+ 13.0 ± 3.0 (+ 15.0 ± 6.0%)	+ 21.0 ± 6.0 (+ 35.0 ± 17.0%)	0.38
	EF (%)	+ 1.4 ± 0.9	+ 6.1 ± 1.5	0.01
LV	EDVi (ml/m^2^) per TTVi (ml/m^2^)	-5.3 ± 2.3 (-8.6 ± 3.0%)	-4.7 ± 1.6 (-6.1 ± 1.8%)	0.28
	ESVi (ml/m^2^) per TTVi (ml/m^2^)	+ 0.4 ± 0.4 (+ 1.7 ± 1.6%)	+ 0.7 ± 0.5 (+ 2.1 ± 2.0%)	0.31
	SVi (ml/m^2^) per TTVi (ml/m^2^)	-5.5 ± 2.5 (-13.9 ± 4.7%)	-5.3 ± 1.7 (-10.2 ± 3.0%)	0.36
	EF (%)	-3.0 ± 1.8	-3.3 ± 1.1	0.47

*EDVi* end-diastolic volume indexed to body surface area, *EF* ejection fraction, *ESVi* end-systolic volume indexed to body surface area, *LV* left ventricular, *RV* right ventricular, *SVi* stroke volume indexed to body surface area, *TTVi* typical tidal volume indexed to body surface area

In comparison to the controls, the RV overload patients showed a smaller increase in RV-SVi, which was accompanied by a mild increase in RV end-systolic volume (RV-ESVi) (Fig. 2, Table 3). In contrast, the RV-ESVi remained almost unchanged with increasing tidal volume in controls (Fig. 2, Table 3). Accordingly, the right RV-EF of the controls increased significantly, whereas the increase in RV-EF in the RV overload patients was substantially lower (Fig. 2, Table 3).

Thus, the main difference between the two groups was a reduced increase in the ejection fraction during inspiration in the RV overload group.

**Fig. 3:**
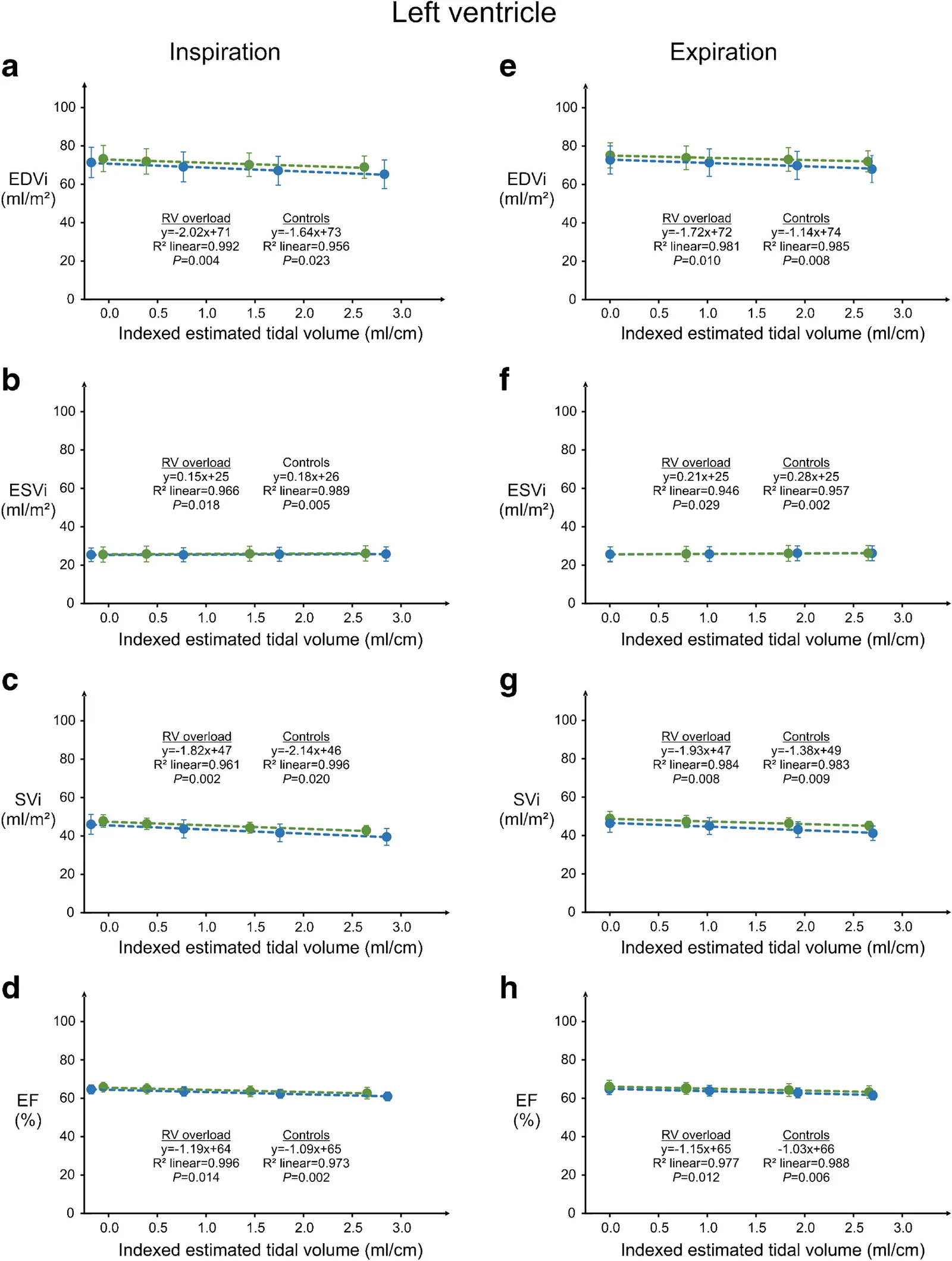
Left ventricular volumes. Mean values (± SD) of left ventricular end-diastolic volume, end-systolic volume, stroke volume, and ejection fraction indexed to the body surface area as a function of the estimated tidal volume indexed to body height in inspiration (**a**–**d**) and expiration (**e**–**h**) for right ventricular overload patients (*blue*) and controls (*green*). Results of the corresponding linear regression analyses are inserted in the graphs. Circles indicate mean value, *whiskers*, SD. *P<0.05 (linear regression). *EDVi* end-diastolic volume indexed to body surface area, *ESVi* end-systolic volume indexed to body surface area, *SVi* stroke volume indexed to body surface area, *EF* ejection fraction, *SD* standard deviation

Linear regression revealed that the respiratory-related changes for RV-EDVi, RV-ESVi, RV-SVi, and RV-EF in RV overload patients and controls were statistically significant during inspiration and expiration (Fig. 3).

In addition to the mean RV volumetry values from RV overload and control patients (Fig. 2, Table 3), detailed RV volumetry results from all patients are presented in Supplementary Material 5.

### Left ventricle

#### Qualitative analysis

The qualitative view of the LV at different time points during the respiratory cycle demonstrates the influence of respiration on LV dimensions. With increasing lung volume, the shape of the LV changes from round to convex at end-diastole in control patients. In chronic RV overload patients, the LV provides a convex shape at end-expiration that even becomes more convex with increasing tidal volume at end-diastole. For details, see Supplementary Material 3 and Supplementary Material 4.

#### Quantitative analysis

Respiratory-dependent LV volume changes were less pronounced and similar in RV overload patients andcontrols (Fig. 3, Table 3). LV-EDVi, LV-SVi, and LV ejection fraction (LV-EF) decreased with increasing tidal volume during inspiration, whereas the LV end-systolic volume (LV-ESVi) showed a small increase (Fig. 3, Table 3).

During expiration, the respiratory influence on LV-EDVi, LV-SVi, and LV-EF was smaller than during inspiration.

Linear regression revealed that the respiratory-related changes for LV-EDVi, LV-ESVi, LV-SVi, and LV-EF in RV overload patients and controls were statistically significant during inspiration and expiration (Fig. 3).

### Frank-Starling relationship

**Fig. 4:**
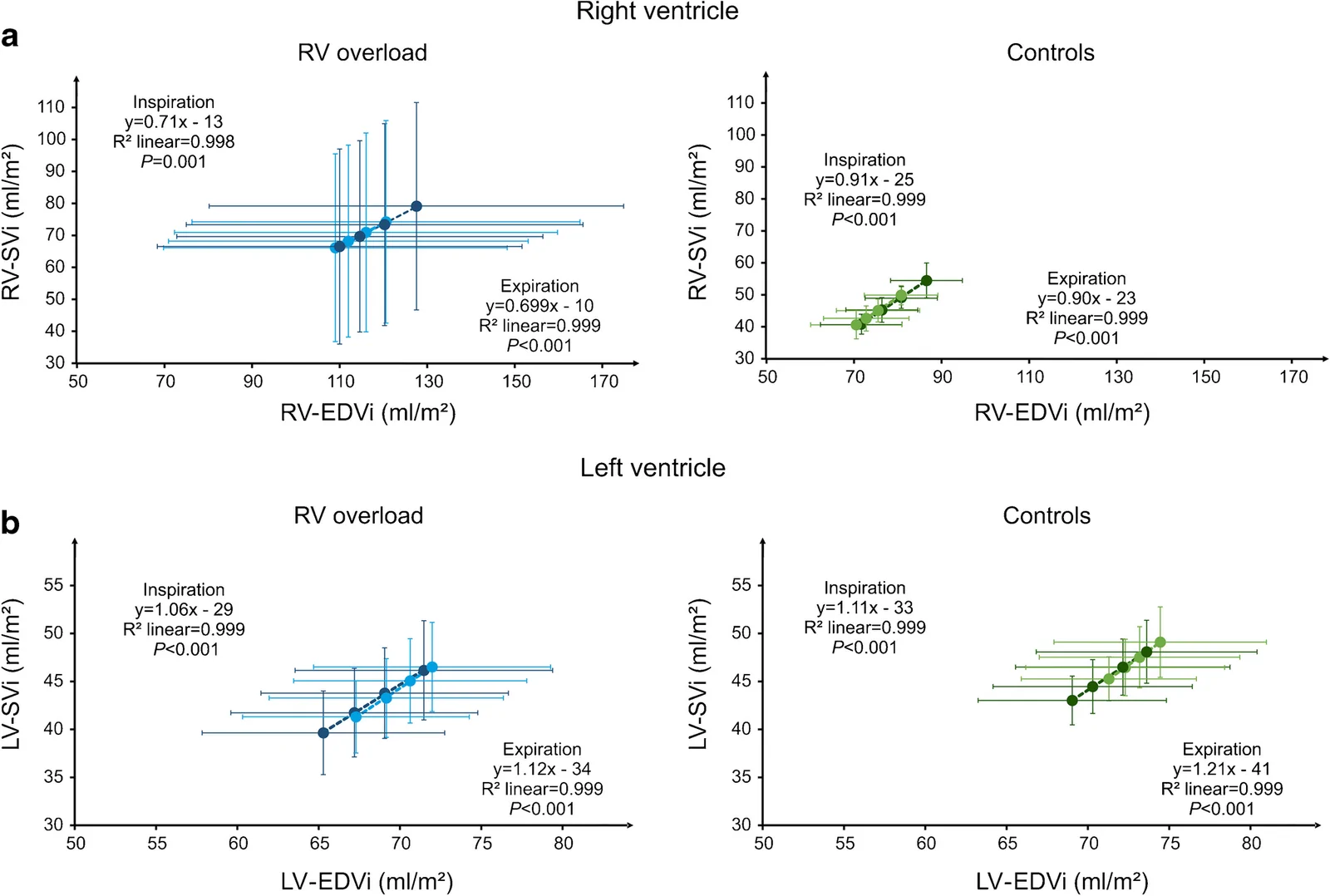
Frank-Starling relationship. Relationship between stroke volume and end-diastolic volume indexed to the body surface area for right patients with ventricle (**a**) and left ventricle (**b**) in inspiration and expiration for right ventricular overload (*blue*) and controls (*green*). Results of the corresponding linear regression analyses are inserted in the graphs. Note, while the right ventricular end-diastolic volume gets larger during inspiration, the left ventricular end-diastolic volume gets larger during expiration. *Circles* indicate mean value, *whiskers* standard deviation. *P<0.05 (linear regression). *LV-EDVi* left ventricular end-diastolic volume indexed to body surface area, *LV-SVi* left ventricular stroke volume indexed to body surface area, *RV-EDVi* right ventricular end-diastolic volume indexed to body surface area, *RV-SVi* right ventricular end-diastolic volume indexed to body surface area

Respiratory-dependent changes of the LV- and RV-EDVi (Δ EDVi >5 ml/m^2^) enabled the analysis of the Frank-Starling relationship. Linear regression revealed a highly significant relationship between SVi and EDVi for RV and the LV during inspiration and expiration (Fig. 4).

The Frank-Starling relationship of the RV provided a significantly reduced slope estimation in the RV overload patients compared to controls in inspiration and expiration (inspiration: RV overload: 0.75 ± 0.11; controls: 0.92 ± 0.02; *P*=0.02; expiration: RV overload: 0.74 ± 0.10; controls: 0.92 ± 0.04; *P*=0.01) (Fig. 4).

In contrast, the slope of the LV showed similar results in the RV overload patients and controls during inspiration and expiration (inspiration: RV overload: 1.05 ± 0.07; controls: 1.12 ± 0.14, expiration: RV overload: 1.05 ± 0.07; controls: 1.13 ± 0.14) (Fig. 4).

## Discussion

The aim of this study was to analyze the modifications of cardiac function induced by breathing in pediatric patients with chronic RV overload. Respiration preferentially modifies the pulmonary circulation. Therefore, right ventricular pathology is more likely to be challenged by breathing maneuvers than left ventricular disorders [11–14]. In addition, right ventricular problems are more frequent in children than in adults [1]. In a previous study in healthy adults, we demonstrated a physiological increase in stroke volume with an increase in ventricular volume, i.e. a physiological Frank-Starling mechanism; whereas RV volumes increased in inspiration, the increase in LV volumes was observed in expiration [14]. The Frank-Starling mechanism, a fundamental law of cardiac physiology, provides an important parameter for the assessment of ventricular function [20, 21]. It describes that with increasing preload, corresponding to an increasing end-diastolic volume, a higher ventricular pressure can be produced, resulting in a higher stroke volume [37]. Molecularly, this mechanism relies on increased sarcomere lengthening, causing a titin-modulated reduction of interfilament lattice spacing, increasing myosin attachment to actin [38]. The increase in RV preload is explained by an increasing venous return to the RV [12–14]. The observed decrease in LV-EDVi with increasing tidal volume has also been demonstrated in previous studies [12–14] and is typically explained by a decrease in LV compliance and an increase in LV afterload [13, 39, 40].

In contrast to our study in healthy volunteers, in this retrospective pediatric study, we did not use MR-compatible spirometry. Similar to other studies in adult [13, 14] and pediatric patients [17, 18], we used the position of the diaphragm for respiratory binning. We generated a calibration curve obtained by MR-compatible spirometry in healthy volunteers to estimate the tidal volume, which is a prerequisite for a fair comparison between patients (see Supplementary Material 1 for further discussion).

The results of the current study extend the findings of our previous study, demonstrating a normal Frank-Starling mechanism of both ventricles in pediatric patients without chronic RV volume overload. Similar to our study in healthy adult volunteers [14], the respiratory influence on ventricular volumes was more pronounced during inspiration than during expiration. Again, breathing influenced the RV significantly more than the LV [12–14]. In our pediatric patients with chronic RV overload, the small decrease in LV-SVi and LV-EF was unaffected, i.e., the Frank-Starling curve of the LV remained physiological. This is explained by the normal size and probable physiological LV preload in both groups [41].

In patients with chronic RV volume overload, the RV-EDVi also increased with increasing tidal volume, especially during inspiration. This indicates that further expansion of the RV was still possible. However, the increase in RV-SVi and RV-EF with increasing tidal volume was significantly reduced indicating an inadequate Frank-Starling mechanism with a significantly decreased slope of the Frank-Starling curve. A flattened or even negative slope of the Frank-Starling curve is a well-described phenomenon in heart failure with dilatation of the LV. This is clinically highly relevant because in these situations additional intravasal fluid could be detrimental, resulting in pulmonary congestion [42]. To predict fluid responsiveness in these patients, dynamic methods such as passive leg raising [43] or a mini-fluid challenge in combination with echocardiography has been proven to be superior to static assessments [44].

Indications for interventions in patients with chronic RV volume overload are still a matter of intense debate. The majority of papers [24] and guidelines [23, 45, 46] propose primarily static parameters, especially the indexed RV-EDV and RV-ESV [23, 24, 45–47]. However, since in other scenarios dynamic parameters have been shown to be superior [44], the simple and physiological respiratory-induced increase in the RV-EDV and the slope of Frank-Starling curve that can be calculated thereof might have the potential to add further information to decide on the timing of interventions.

The role of diffuse fibrosis in the pathomechanism of RV failure in patients with RV pressure overload is well established [48]. However, its role in chronic RV volume overload is still unclear. Signs of diffuse fibrosis as indicated by an increase in T1 relaxation times have been described by Cochet et al. [49] in young adults and Yim et al. [50] in children with repaired tetralogy of Fallot with chronic volume overload. However, the experimental study by Hagdorn et al. in Wistar rats with chronic RV volume overload could not demonstrate myocardial fibrosis [41]. It is difficult to base our observations on the presence of fibrosis, whichwould not flatten the slope of the Frank-Starling curve. Rather, the reduced distensibility of the fibrotic ventricular wall would prevent the Frank-Starling mechanism from becoming effective [51]. An alternative explanation would be an increased stiffness of sarcomeres due to an increase in the stiffer titin isoform N2B, which may be related to RV insufficiency [41]. Unfortunately, our study cannot provide information on the underlying pathophysiology.

Despite the preliminary nature of our observations, we are not aware of any study that has previously investigated the Frank-Starling relationship in pediatric patients with congenital heart disease under physiological conditions non-invasively. The present work describes first experiences with the decrease in the slope of the Frank-Starling curve in pediatric patients with chronic RV volume overload. This observation might represent a valuable dynamic parameter to measure consequences of RV volume overload. However, several limitations must be considered.

First, the number of patients is relatively small and the mean age of the patients, height, and body weight were on average higher in patients without cardiac abnormalities than in the chronic RV overload group. In addition, patients with chronic RV volume overload had on average higher respiratory rates. More patients are required to test and verify the clinical relevance of the results. Second, the retrospective design precludes any conclusions on the prognostic information of the Frank-Starling curve. A prospective comparison of MRI data before and after an intervention that reduces RV overload would be needed to judge the prognostic information provided by a disturbed Frank-Starling mechanism. Third, tidal volumes were based on an estimate obtained via a calibration curve in healthy adults. A direct MR-compatible spirometry is likely to be more precise. Fourth, postprocessing is extremely time-consuming (~ 3 days) because automatic contouring of the RV requires manual confirmation.

Future prospective studies should include a larger number and a more homogenous population of patients. For example, based on previous studies, left-to-right shunt lesions, pulmonary regurgitation after pulmonary stenosis, and repaired tetralogy of Fallot can be expected to behave differently [52, 53]. In addition, well-matched controls and follow-up investigations after interventions will be needed to confirm the results and assess the prognostic relevance of the Frank-Starling curve. Additional real-time phase-contrast flow measurements will improve data quality. MR-compatible spirometry will provide exact respiratory volumes and carbon dioxide measurements for standardization. Late gadolinium enhancement or T1 mapping might help to understand the underlying pathomechanism of the disturbed Frank-Starling mechanism. Finally, artificial intelligence could help to improve and accelerate image analysis, particularly contouring; this would ease integration of this parameter into routine clinical practice.

## Conclusion

Real-time MRI during free breathing in combination with respiratory binning allows the assessment of respiratory-induced effects on ventricular volume and function. First experience in pediatric patients with chronic RV volume overload demonstrates an impairment of the Frank-Starling relationship of the RV that might provide a new parameter to optimize timing of interventions.

## Supplementary Information

Below is the link to the electronic supplementary material.

Supplementary file1 (DOCX 340 KB)

Supplementary file2 (MP4 2077 KB)

Supplementary file3 (MP4 4596 KB)

Supplementary file4 (MP4 4662 KB)

Supplementary file5 (DOCX 52.0 KB)

## Data Availability

The code and an example of a data set that serves to demonstrate the code are openly available: https://github.com/MPR-UKD/RT-MRI-Respiratory-Cardiac-Function-CHD. All other datasets are available from the corresponding author on reasonable request.
