# Supplementary material for: First experience with real-time magnetic resonance imaging-based investigation of respiratory influence on cardiac function in pediatric congenital heart disease patients with chronic right ventricular volume overload
Source: Pediatr Radiol. 2023 Oct 5;53(13):2608–21. doi: 10.1007/s00247-023-05765-9 (PMC10698081; doi:10.1007/s00247-023-05765-9)
Supplement: Supplementary file 1 — Supplementary file1 (DOCX 340 KB) [file 247_2023_5765_MOESM1_ESM.docx]

**Supplementary material 1**

**Calibration study to estimate tidal volume from
respiratory-induced cardiac movement
using magnetic resonance-compatible spirometry**

Introduction:

Spontaneous breathing modifies cardiac function [1]. The driving force behind this process is primarily the alteration of the transmural pressure, which increases with increasing lung volume [1]. Quantifying lung volume is a prerequisite for a comparison between patients of different ages, lengths, and varying depths of respiration. However, the magnetic resonance imaging (MRI) protocol in our main study did not yet include the gold standard to measure lung volumes, i.e., magnetic resonance-compatible spirometry [2, 3].

In our main study, we used the signal intensity (SI) of a region of interest (ROI) close to the diaphragm to monitor respiration. This technique was well suited to perform binning. However, although the SI of these ROIs also mirror lung volume, standardization of this method turned out to be very difficult. In contrast, the position of the heart could be defined very precisely and showed a good correlation with lung volume.

For this reason, we conducted this separate calibration study. The aim of this calibration study was to use the gold standard, i.e. magnetic resonance-compatible spirometry to estimate the tidal volumes from the respiratory-modified cardiac position.

This information was then used in the main study to perform a *bona fide* quantification of the tidal volumes for our patients, thus allowing to compare the cardiovascular effects of spontaneous breathing.

Methods:

Healthy adult volunteers (n=8, 3 male / 5 female), age 32 ± 12 years, body height 176 ± 11 cm, body weight 68 ± 11kg) underwent cardiac real-time MRI during free-breathing in combination with magnetic resonance-compatible spirometry as described previously [2, 3].

Real-time MRI of a midventricular slice in combination with information on tidal volume and respiratory phase (e.g., inspiration/expiration) obtained by magnetic resonance-compatible spirometry was used to determine the relationship between the heart position and tidal volume during a defined time point of the cardiac cycle (e.g., end-diastole).

The displacement of the heart, measured by the midpoint P(m) between the superior and inferior right ventricular insertion point (P1, P2) (Supplementary Material 1: Fig. S1), was determined in a midventricular slice in all respiratory classes see (Supplementary Material 2). The change in tidal volume and the corresponding P(m) displacement in superior (-left-anterior) to inferior (-right-posterior) direction are shown in Supplementary Material 1: Table S1.

Results:

A significant linear correlation existed between the increase in tidal volume as a function of heart position in superior (-left-anterior) to inferior (-right-posterior) direction during normal breathing (Supplementary Material 1: Fig. S1). Only during deep breathing was the significance level of a quadratic correlation superior to the linear correlation (Supplementary Material 1: Fig. S1).

The mean tidal volume during normal breathing was 2.31 ± 2.45 ml/cm body length. The mean movement of the heart during normal breathing was 0.02 ± 0.01 mm/ml tidal volume.

Discussion, imitations and future perspective:

The results demonstrate a linear relationship between cardiac movement and tidal volume for normal breathing. During deep breathing, the displacement of the heart becomes relatively smaller.

Previous studies [1] strongly suggest a correlation between tidal volume and cardiovascular effects of spontaneous breathing. Therefore, we can assume that the estimated tidal volume is relevant for the observed heart-lung interaction.

Since lung volumes are typically referenced to body length [4], we indexed our cardiovascular data to a “typical tidal volume referenced to body length” (TTVi) during spontaneous breathing to allow a fair comparison between patients. We defined a TTVi of 3 ml/cm for indexing, which is close to the average tidal volume in the RV patients and controls.

Although the described method is completely non-invasive, important limitations remain. First, even if there is a correlation between the tidal volume estimated from respiratory movements, these data cannot replace measurements of the lung volumes provided by spirometry. Second, the driving force for the blood flow into the right heart during inspiration is not the lung volume itself but the transmural pressure, i.e. the difference between the pleural and the right atrial pressure. Therefore, even if there is a close relationship between transmural pressure and tidal volume, it will remain a surrogate parameter [1] .

We have shown previously, that magnetic resonance-compatible spirometry during real-time MRI is well tolerated [3]. In the future, this method should help to standardize spontaneous breathing with respect to tidal volume, respiratory rate and carbon dioxide measurements.

Conclusion:

Tidal volumes can be estimated from respiratory-induced cardiac movement.

Supplementary Material: Figure S1

*Calibration curves.* Tidal volume calculation based on calibration curves obtained in healthy volunteers (n=8) with magnetic resonance-compatible spirometry during normal breathing (dotted line) and deep breathing (solid line). **a** Definition of the position of the heart by the midpoint P(m) between the superior and inferior right ventricular insertion points (P1, P2). **b** Calibration curves demonstrating the relationship between the movement of the midpoint P(m) in superior (-left-anterior) to inferior (-right-posterior) direction and the height-indexed tidal volume of eight healthy volunteers (dots in different colors) at end-diastole during normal breathing (dark yellow line; linear regression) and deep breathing (black curve; quadratic regression). The gray box marks the values in the range of normal breathing up to 3 ml/cm.


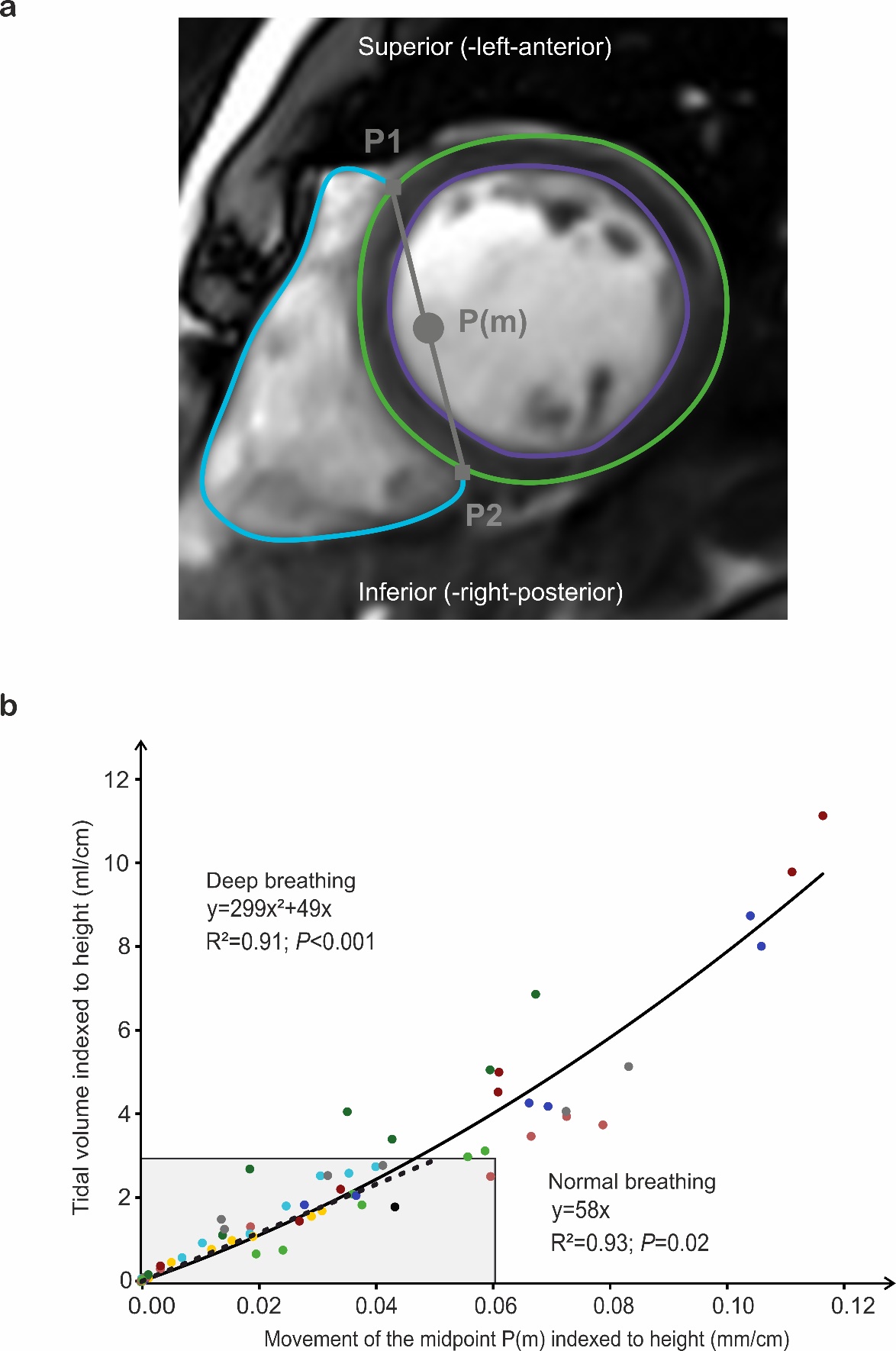


| Subject | Respiratory class | | Tidal volume (ml/cm) | Heart position (y-axis) (mm) | | | Tidal volume change  (ml/cm) | P(m) movement  (mm/cm) | P(m)  movement (mm/ml) |
| --- | --- | --- | --- | --- | --- | --- | --- | --- | --- |
|  | **Respiratory phase** | **Volume class** |  | **P1** | **P2** | **P(m)** |  |  |  |
| 01 | Inspiration | High-maximum | 0.29 | 119.79 | 190.09 | 154.94 | 0.38 | 0.00 | 0.01 |
|  |  | Medium-high | 1.04 | 122.04 | 192.90 | 157.47 | 1.13 | 0.02 | 0.02 |
|  |  | Low-medium | 2.44 | 124.85 | 194.50 | 159.68 | 2.53 | 0.03 | 0.01 |
|  |  | Minimum-low | 2.49 | 125.98 | 195.15 | 160.57 | 2.57 | 0.04 | 0.01 |
|  | Expiration | High-maximum | -0.08 | 118.66 | 189.53 | 154.10 | 0.00 | 0.00 | - |
|  |  | Medium-high | 0.84 | 121.48 | 190.65 | 156.07 | 0.92 | 0.01 | 0.01 |
|  |  | Low-medium | 1.70 | 123.73 | 193.46 | 158.60 | 1.79 | 0.02 | 0.01 |
|  |  | Minimum-low | 2.64 | 126.54 | 196.27 | 161.41 | 2.72 | 0.04 | 0.01 |
| 02 | Inspiration | High-maximum | 0,00 | 118.66 | 192.90 | 155.78 | 0.19 | 0.00 | 0.02 |
|  |  | Medium-high | 1.58 | 125.41 | 200.77 | 163.09 | 1.77 | 0.04 | 0.02 |
|  |  | Low-medium | 2.33 | 126.66 | 205.52 | 166.09 | 2,52 | 0.06 | 0.02 |
|  |  | Minimum-low | 3.54 | 130.47 | 208.65 | 169.56 | 3.73 | 0.08 | 0.02 |
|  | Expiration | High-maximum | -0.19 | 118.10 | 192.34 | 155.22 | 0.00 | 0.00 | - |
|  |  | Medium-high | 1.10 | 122.04 | 195.15 | 158.60 | 1.30 | 0.02 | 0.01 |
|  |  | Low-medium | 3.27 | 128.79 | 205.83 | 167.31 | 3.47 | 0.07 | 0.02 |
|  |  | Minimum-low | 3.76 | 129.91 | 206.96 | 168.44 | 3.96 | 0.07 | 0.02 |
| 03 | Inspiration | High-maximum | 0.03 | 165.42 | 231.14 | 198.28 | 0.02 | 0.00 | 0.01 |
|  |  | Medium-high | 1.25 | 167.20 | 234.52 | 200.86 | 1.23 | 0.01 | 0.01 |
|  |  | Low-medium | 2.77 | 175.02 | 236.77 | 205.90 | 2.76 | 0.04 | 0.01 |
|  |  | Minimum-low | 4.04 | 183.90 | 239.58 | 211.74 | 4.02 | 0.07 | 0.02 |
|  | Expiration | High-maximum | 0.02 | 165.34 | 231.14 | 198.24 | 0.00 | 0.00 | - |
|  |  | Medium-high | 1.49 | 167.59 | 233.95 | 200.77 | 1.47 | 0.01 | 0.01 |
|  |  | Low-medium | 2.54 | 172.65 | 235.64 | 204.15 | 2.53 | 0.03 | 0.01 |
|  |  | Minimum-low | 5.14 | 185.03 | 242.39 | 213.71 | 5.12 | 0.08 | 0.02 |
| 04 | Inspiration | High-maximum | 0.00 | 119.79 | 179.20 | 149.50 | 0.07 | 0.00 | 0.02 |
|  |  | Medium-high | 0.44 | 121.48 | 179.40 | 150.44 | 0.51 | 0.01 | 0.01 |
|  |  | Low-medium | 1.01 | 123.73 | 181.09 | 152.41 | 1,08 | 0.02 | 0.02 |
|  |  | Minimum-low | 1.48 | 124.85 | 183.34 | 154.10 | 1.55 | 0.03 | 0.02 |
|  | Expiration | High-maximum | -0.07 | 119.79 | 178.84 | 149.32 | 0.00 | 0.00 | - |
|  |  | Medium-high | 0.64 | 122.04 | 180.53 | 151.29 | 0.71 | 0.01 | 0.02 |
|  |  | Low-medium | 0.90 | 122.60 | 181.09 | 151.85 | 0.97 | 0.02 | 0.02 |
|  |  | Minimum-low | 1.62 | 124.85 | 183.90 | 154.38 | 1.69 | 0.03 | 0.02 |
| 05 | Inspiration | High-maximum | 0.08 | 130.47 | 191.24 | 160.86 | 0.00 | 0.00 | 0.02 |
|  |  | Medium-high | 2.75 | 132.72 | 195.71 | 164.22 | 2.66 | 0.02 | 0.01 |
|  |  | Low-medium | 4.14 | 134.41 | 200.21 | 167.31 | 4.07 | 0.04 | 0.01 |
|  |  | Minimum-low | 6.92 | 138.91 | 207.52 | 173.22 | 6.85 | 0.07 | 0.01 |
|  | expiration | High-maximum | 0.07 | 130.47 | 191.21 | 160.84 | 0.00 | 0.00 | - |
|  |  | Medium-high | 1.17 | 132.16 | 194.59 | 163.38 | 1.10 | 0.01 | 0.01 |
|  |  | Low-medium | 3.47 | 135.54 | 201.90 | 168.72 | 3.40 | 0.04 | 0.01 |
|  |  | Minimum-low | 5.13 | 137.79 | 205.83 | 171.81 | 5.06 | 0.06 | 0.01 |
| 06 | inspiration | High-maximum | 0.03 | 129.35 | 193.57 | 161.46 | 0.02 | 0.00 | 0.02 |
|  |  | Medium-high | 1.84 | 134.97 | 196.84 | 165.91 | 1.83 | 0.03 | 0.02 |
|  |  | Low-medium | 4.21 | 141.72 | 203.59 | 172.66 | 4.19 | 0.07 | 0.02 |
|  |  | Minimum-low | 8.01 | 146.22 | 210.90 | 178.56 | 8.00 | 0.11 | 0.01 |
|  | Expiration | High-maximum | 0.01 | 129.35 | 193.46 | 161.41 | 0.00 | 0.00 | - |
|  |  | Medium-high | 2.05 | 136.66 | 197.96 | 167.31 | 2.04 | 0.04 | 0.02 |
|  |  | Low-medium | 4.27 | 140.06 | 204.15 | 172.11 | 4.26 | 0.07 | 0.02 |
|  |  | Minimum-low | 8.75 | 144.97 | 211.46 | 178.22 | 8.74 | 0.10 | 0.01 |
| 07 | Inspiration | High-maximum | 0.13 | 117.95 | 164.80 | 141.38 | 0.28 | 0.00 | 0.01 |
|  |  | Medium-high | 1.29 | 118.66 | 171.53 | 145.10 | 1.44 | 0.03 | 0.02 |
|  |  | Low-medium | 4.35 | 120.91 | 179.96 | 150.44 | 4.50 | 0.06 | 0.01 |
|  |  | Minimum-low | 10.98 | 129.35 | 188.96 | 159.16 | 11.13 | 0.12 | 0.01 |
|  | Expiration | High-maximum | -0.15 | 117.54 | 164.22 | 140.88 | 0.00 | 0.00 | - |
|  |  | Medium-high | 2.05 | 118.66 | 173.78 | 146.22 | 2.20 | 0.03 | 0.02 |
|  |  | Low-medium | 4.84 | 120.35 | 180.53 | 150.44 | 4.99 | 0.06 | 0.01 |
|  |  | Minimum-low | 9.65 | 128.79 | 187.84 | 158.32 | 9.80 | 0.11 | 0.01 |
| 08 | Inspiration | High-maximum | 0.00 | 134.97 | 204.73 | 169.85 | 0.00 | 0.00 | 0.02 |
|  |  | Medium-high | 0.65 | 137.22 | 209.77 | 173.50 | 0.65 | 0.02 | 0.03 |
|  |  | Low-medium | 1.82 | 140.60 | 213.15 | 176.88 | 1.82 | 0.04 | 0.02 |
|  |  | Minimum-low | 2.98 | 141.72 | 218.77 | 180.25 | 2.99 | 0.06 | 0.02 |
|  | Expiration | High-maximum | 0.00 | 134.97 | 204.71 | 169.84 | 0.00 | 0.00 | - |
|  |  | Medium-high | 0.73 | 137.79 | 210.90 | 174.35 | 0.73 | 0.02 | 0.03 |
|  |  | Low-medium | 2.07 | 140.04 | 213.15 | 176.60 | 2.07 | 0.04 | 0.02 |
|  |  | Minimum-low | 3.12 | 142.85 | 218.77 | 180.81 | 3.12 | 0.06 | 0.02 |
| Mean ± SD | **-** |  | **2.31 ± 2.45** | **134.41 ± 16.57** | **199.83 ± 18.13** | **167.12 ± 16.98** | **2.35 ± 2.45** | **0.03 ± 0.03** | **0.02 ± 0.01** |

Supplementary Material: Table S1: Tidal volume and heart displacement]

Tidal volumes, corresponding heart position and respiratory induced cardiac movement from eight healthy volunteers as basis for the calculation of the calibration curves. P(m) = P (midpoint), SD = standard deviation.

Supplementary Material 1: Table S2: Tidal volume estimation RV overload patients

| Patient | Respiratory class | | SI (mean) | Mean heart position (Pm) (y-axis) (mm) | Mean heart movement P(m) (y-axis) (mm) | Mean heart movement P(m) indexed to height  (mm/cm) | Estimated tidal volume (ml) | Estimated tidal volume indexed to height (ml/cm) |
| --- | --- | --- | --- | --- | --- | --- | --- | --- |
|  | Respiratory phase | Volume class |  |  |  |  |  |  |
| 01 | Inspiration | High-maximum | 347 ± 97 | 220 ± 2.0 | 5.3 | 0.04 | 306 | 2.4 |
|  |  | Medium-high | 377 ± 83 | 218 ± 1.7 | 3.8 | 0.03 | 219 | 1.7 |
|  |  | Low-medium | 415 ± 86 | 216 ± 1.2 | 1.6 | 0.01 | 92 | 0.7 |
|  |  | Minimum-low | 444 ± 83 | 214 ± 2.3 | -0.6 | 0.00 | -33 | -0.3 |
|  | Expiration | High-maximum | 367 ± 90 | 219 ± 1.8 | 4.6 | 0.04 | 268 | 2.1 |
|  |  | Medium-high | 388 ± 83 | 217 ± 1.6 | 3.2 | 0.02 | 183 | 1.4 |
|  |  | Low-medium | 415 ± 88 | 215 ± 1.9 | 1.2 | 0.01 | 67 | 0.5 |
|  |  | Minimum-low | 441 ± 83 | 214 ± 1.8 | 0.0 | 0.00 | 0 | 0.0 |
| 02 | Inspiration | High-maximum | 296 ± 22 | 251 ± 1.1 | 4.4 | 0.03 | 254 | -0.2 |
|  |  | Medium-high | 443 ± 95 | 249 ± 1.3 | 2.7 | 0.02 | 158 | 0.4 |
|  |  | Low-medium | 572 ± 155 | 247 ± 1.8 | 1.0 | 0.01 | 58 | 1.2 |
|  |  | Minimum-low | 688 ± 215 | 246 ± 2.0 | -0.4 | 0.00 | -25 | 2.0 |
|  | Expiration | High-maximum | 377 ± 80 | 250 ± 1.1 | 3.9 | 0.03 | 224 | 1.7 |
|  |  | Medium-high | 488 ± 178 | 250 ± 0.5 | 3.4 | 0.03 | 196 | 1.5 |
|  |  | Low-medium | 567 ± 161 | 249 ± 1.5 | 2.5 | 0.02 | 144 | 1.1 |
|  |  | Minimum-low | 664 ± 213 | 246 ± 1.7 | 0.0 | 0.00 | 0 | 0.0 |
| 03 | Inspiration | High-maximum | 214 ± 27 | 225 ± 1.4 | 6.0 | 0.05 | 349 | 2.6 |
|  |  | Medium-high | 404 ± 94 | 222 ± 1.3 | 3.3 | 0.02 | 190 | 1.4 |
|  |  | Low-medium | 592 ± 186 | 221 ± 1.1 | 1.9 | 0.01 | 112 | 0.8 |
|  |  | Minimum-low | 809 ± 255 | 218 ± 1.7 | -1.0 | -0.01 | -59 | -0.4 |
|  | Expiration | High-maximum | 244 ± 33 | 225 ± 1.3 | 5.8 | 0.04 | 337 | 2.5 |
|  |  | Medium-high | 388 ± 112 | 223 ± 0.8 | 4.3 | 0.03 | 251 | 1.9 |
|  |  | Low-medium | 619 ± 161 | 222 ± 0.9 | 3.1 | 0.02 | 180 | 1.4 |
|  |  | Minimum-low | 821 ± 278 | 219 ± 2.0 | 0.0 | 0.00 | 0 | 0.0 |
| 04 | Inspiration | High-maximum | 289 ± 35 | 228 ± 4.9 | 6.2 | 0.05 | 361 | 3.1 |
|  |  | Medium-high | 492 ± 74 | 225 ± 5.4 | 3.5 | 0.03 | 203 | 1.7 |
|  |  | Low-medium | 789 ± 285 | 223 ± 5.0 | 1.6 | 0.01 | 91 | 0.8 |
|  |  | Minimum-low | 1001 ± 331 | 222 ± 5.4 | 0.1 | 0.00 | 8 | 0.1 |
|  | Expiration | High-maximum | 295 ± 23 | 228 ± 5.1 | 5.8 | 0.05 | 334 | 2.9 |
|  |  | Medium-high | 546 ± 158 | 225 ± 4.9 | 2.9 | 0.02 | 166 | 1.4 |
|  |  | Low-medium | 800 ± 303 | 224 ± 4.9 | 1.7 | 0.01 | 97 | 0.8 |
|  |  | Minimum-low | 1008 ± 347 | 222 ± 5.1 | 0.0 | 0.00 | 00 | 0.0 |
| 05 | Inspiration | High-maximum | 186 ± 22 | 225 ± 1.4 | 5.9 | 0.05 | 365 | 2.7 |
|  |  | Medium-high | 377 ± 45 | 223 ± 1.4 | 3.2 | 0.03 | 179 | 1.3 |
|  |  | Low-medium | 697 ± 94 | 220 ± 1.3 | 0.4 | 0.01 | 18 | 0.1 |
|  |  | Minimum-low | 791 ± 118 | 219 ± 1.2 | -0.4 | 0.00 | -21 | -0.2 |
|  | Expiration | High-maximum | 211 ± 40 | 225 ± 1.3 | 5.5 | 0.05 | 338 | 2.5 |
|  |  | Medium-high | 380 ± 103 | 223 ± 1.7 | 3.8 | 0.02 | 218 | 1.6 |
|  |  | Low-medium | 664 ± 111 | 220 ± 1.1 | 0.9 | 0.01 | 47 | 0.3 |
|  |  | Minimum-low | 783 ± 112 | 219 ± 1.1 | 0.0 | 0.00 | 00 | 0.0 |
| 06 | Inspiration | High-maximum | 537 ± 162 | 206 ± 3.5 | 11.5 | 0.07 | 667 | 4.3 |
|  |  | Medium-high | 867 ± 335 | 202 ± 2.8 | 8.3 | 0.05 | 479 | 3.1 |
|  |  | Low-medium | 1205 ± 213 | 199 ± 3.8 | 4.5 | 0.03 | 259 | 1.7 |
|  |  | Minimum-low | 1445 ± 300 | 194 ± 1.1 | -0.4 | 0.00 | -21 | -0.1 |
|  | Expiration | High-maximum | 515 ± 198 | 206 ± 3.1 | 11.9 | 0.08 | 689 | 4.4 |
|  |  | Medium-high | 886 ± 325 | 204 ± 2.9 | 9.8 | 0.06 | 571 | 3.7 |
|  |  | Low-medium | 1216 ± 242 | 199 ± 3.9 | 5.2 | 0.03 | 303 | 2.0 |
|  |  | Minimum-low | 1495 ± 316 | 194 ± 0.9 | 0.0 | 0.00 | 0 | 0.0 |
| Mean ± SD | Inspiration | High-maximum | 311 ± 114 | 226 ± 13 | 6.6 ± 2.3 | 0.05 ± 0.01 | 384 ± 132 | 2.8 ± 0.7 |
|  |  | Medium-high | 493 ± 172 | 223 ± 14 | 4.1 ± 1.9 | 0.03 ± 0.01 | 238 ± 110 | 1.8 ± 0.6 |
|  |  | Low-medium | 712 ± 249 | 221 ± 14 | 1.8 ± 1.3 | 0.01 ± 0.01 | 105 ± 75 | 0.8 ± 0.5 |
|  |  | Minimum-low | 863 ± 309 | 219 ± 15 | -0.4 ± 0.3 | 0.00 ± 0.00 | -25 ± 20 | -0.2 ± 0.2 |
|  | Expiration | High-maximum | 335 ± 100 | 225 ± 13 | 6.2 ± 2.4 | 0.05 ± 0.02 | 365 ± 151 | 2.7 ± 0.9 |
|  |  | Medium-high | 513 ± 178 | 224 ± 14 | 4.6 ± 2.4 | 0.03 ± 0.01 | 264 ± 140 | 1.9 ± 0.8 |
|  |  | Low-medium | 713 ± 252 | 222 ± 15 | 2.4 ± 1.5 | 0.02 ± 0.01 | 140 ± 86 | 1.0 ± 0.5 |
|  |  | Minimum-low | 869 ± 328 | 219 ± 15 | 0.0 ± 0.0 | 0.00 ± 0.00 | 0.0 ± 0.0 | 0.0 ± 0.0 |
| Mean ± SD all respiratory classes | | | **601 ± 206** | **222 ± 2.5** | **3.2 ± 2.5** | **0.02 ± 0.02** | **184 ± 145** | **1.4 ± 0.8** |

Mean SI values, the corresponding heart position and respiratory induced cardiac movement of six RV overload patients as basis for the estimation of tidal volumes.
P(m) = P (midpoint), SD = standard deviation, SI = signal intensity.

Supplementary Material 1: Table S3: Tidal volume estimation controls

| Patient | Respiratory class | | SI (mean) | Mean heart position (Pm) (y-axis) (mm) | Mean heart movement P(m) (y-axis) (mm) | Mean heart movement P(m) indexed to height  (mm/cm) | Estimated tidal volume (ml) | Estimated tidal volume indexed to height (ml/cm) |
| --- | --- | --- | --- | --- | --- | --- | --- | --- |
|  | Respiratory phase | Tidal volume |  |  |  |  |  |  |
| 01 | Inspiration | High-maximum | 118 ± 15 | 278 ± 2.4 | 8.4 | 0.05 | 485 | 3.2 |
|  |  | Medium-high | 276 ± 21 | 274 ± 1.5 | 4.3 | 0.03 | 251 | 1.6 |
|  |  | Low-medium | 516 ± 89 | 271 ± 1.3 | 0.8 | 0.01 | 47 | 0.3 |
|  |  | Minimum-low | 615 ± 114 | 270 ± 1.3 | -0.3 | 0.00 | -18 | -0.1 |
|  | Expiration | High-maximum | 131 ± 22 | 278 ± 2.4 | 7.9 | 0.05 | 458 | 3.0 |
|  |  | Medium-high | 264 ± 53 | 275 ± 1.9 | 5.3 | 0.03 | 308 | 2.0 |
|  |  | Low-medium | 520 ± 87 | 272 ± 1.7 | 2.2 | 0.01 | 125 | 0.8 |
|  |  | Minimum-low | 617 ± 133 | 270 ± 1.6 | 0.0 | 0.00 | 0 | 0.0 |
| 02 | Inspiration | High-maximum | 174 ± 58 | 223 ± 5.8 | 10.2 | 0.06 | 589 | 3.6 |
|  |  | Medium-high | 335 ± 133 | 219 ± 2.1 | 6.3 | 0.04 | 368 | 2.2 |
|  |  | Low-medium | 717 ± 234 | 215 ± 3.3 | 3.1 | 0.02 | 177 | 1.1 |
|  |  | Minimum-low | 982 ± 98 | 213 ± 2.2 | 0.2 | 0.00 | 9 | 0.1 |
|  | Expiration | High-maximum | 152 ± 58 | 224 ± 4.8 | 11.4 | 0.07 | 662 | 4.0 |
|  |  | Medium-high | 362 ± 116 | 222 ± 5.7 | 9.1 | 0.06 | 527 | 3.2 |
|  |  | Low-medium | 737 ± 133 | 217 ± 3.8 | 4.4 | 0.03 | 253 | 1.5 |
|  |  | Minimum-low | 980 ± 81 | 212 ± 1.8 | 0.0 | 0.00 | 0 | 0.0 |
| 03 | Inspiration | High-maximum | 301 ± 62 | 234 ± 4.3 | 8.1 | 0.05 | 473 | 2.9 |
|  |  | Medium-high | 755 ± 258 | 229 ± 3.7 | 2.7 | 0.02 | 159 | 1.0 |
|  |  | Low-medium | 1275 ± 526 | 227 ± 2.9 | 0.4 | 0.00 | 21 | 0.1 |
|  |  | Minimum-low | 1582 ± 494 | 226 ± 2.7 | -0.4 | 0.00 | -24 | -0.1 |
|  | Expiration | High-maximum | 291 ± 42 | 235 ± 6.0 | 8.4 | 0.05 | 485 | 2.9 |
|  |  | Medium-high | 603 ± 233 | 231 ± 3.9 | 5.3 | 0.03 | 305 | 1.9 |
|  |  | Low-medium | 1241 ± 492 | 228 ± 3.0 | 2.0 | 0.01 | 114 | 0.7 |
|  |  | Minimum-low | 1613 ± 486 | 226 ± 2.5 | 0.0 | 0.00 | 0 | 0.0 |
| 04 | Inspiration | High-maximum | 183 ± 54 | 239 ± 0.8 | 6.0 | 0.03 | 349 | 1.9 |
|  |  | Medium-high | 320 ± 107 | 237 ± 0.9 | 3.5 | 0.02 | 204 | 1.1 |
|  |  | Low-medium | 626 ± 218 | 235 ± 1.3 | 1.2 | 0.01 | 71 | 0.4 |
|  |  | Minimum-low | 739 ± 239 | 233 ± 1.8 | 0.2 | 0.00 | 11 | 0.1 |
|  | Expiration | High-maximum | 197 ± 50 | 239 ± 1.1 | 5.4 | 0.03 | 312 | 1.7 |
|  |  | Medium-high | 335 ± 95 | 237 ± 1.5 | 3.5 | 0.02 | 202 | 1.1 |
|  |  | Low-medium | 578 ± 206 | 235 ± 1.0 | 1.7 | 0.01 | 97 | 0.5 |
|  |  | Minimum-low | 742 ± 252 | 233 ± 1.2 | 0.0 | 0.00 | 0 | 0.0 |
| 05 | Inspiration | High-maximum | 969 ± 224 | 238 ± 2.3 | 4.6 | 0.03 | 267 | 1.6 |
|  |  | Medium-high | 1105 ± 198 | 236 ± 2.4 | 2.7 | 0.02 | 157 | 0.9 |
|  |  | Low-medium | 1373 ± 211 | 235 ± 2.8 | 0.9 | 0.01 | 53 | 0.3 |
|  |  | Minimum-low | 1648 ± 156 | 233 ± 3.3 | -0.2 | 0.00 | -12 | -0.1 |
|  | Expiration | High-maximum | 974 ± 209 | 238 ± 2.1 | 4.7 | 0.03 | 271 | 1.6 |
|  |  | Medium-high | 1174 ± 264 | 237 ± 3.0 | 3.3 | 0.02 | 193 | 1.1 |
|  |  | Low-medium | 1498 ± 164 | 235 ± 3.1 | 1.5 | 0.01 | 86 | 0.5 |
|  |  | Minimum-low | 1640 ± 160 | 234 ± 3.4 | 0.0 | 0.00 | 0 | 0.0 |
| 06 | Inspiration | High-maximum | 333 ± 34 | 243 ± 3.3 | 7.3 | 0.05 | 424 | 2.7 |
|  |  | Medium-high | 425 ± 51 | 241 ± 4.3 | 4.8 | 0.03 | 280 | 1.8 |
|  |  | Low-medium | 595 ± 127 | 236 ± 5.5 | 0.3 | 0.00 | 16 | 0.1 |
|  |  | Minimum-low | 673 ± 185 | 236 ± 5.6 | -0.4 | 0.00 | -23 | -0.1 |
|  | Expiration | High-maximum | 353 ± 25 | 243 ± 3.7 | 7.2 | 0.05 | 416 | 2.7 |
|  |  | Medium-high | 468 ± 94 | 241 ± 4.5 | 4.6 | 0.03 | 265 | 1.7 |
|  |  | Low-medium | 567 ± 106 | 238 ± 5.5 | 1.7 | 0.01 | 97 | 0.6 |
|  |  | Minimum-low | 660 ± 168 | 236 ± 5.7 | 0.0 | 0.00 | 0 | 0.0 |
| Mean ± SD | Inspiration | High-maximum | 346 ± 288 | 243 ± 17 | 7.4 ± 1.8 | 0.05 ± 0.01 | 430 ± 103 | 2.6 ± 0.7 |
|  |  | Medium-high | 536 ± 300 | 239 ± 17 | 4.1 ± 1.3 | 0.02 ± 0.01 | 236 ± 74 | 1.4 ± 0.5 |
|  |  | Low-medium | 850 ± 341 | 236 ± 17 | 1.1 ± 0.9 | 0.01 ± 0.01 | 64 ± 54 | 0.4 ± 0.3 |
|  |  | Minimum-low | 1040 ± 423 | 235 ± 17 | -0.2 ± 0.2 | 0.00 ± 0.00 | -9 ± 14 | -0.1 ± 0.1 |
|  | Expiration | High-maximum | 350 ± 290 | 243 ± 17 | 7.5 ± 2.2 | 0.05 ± 0.01 | 434 ± 127 | 2.7 ± 0.8 |
|  |  | Medium-high | 534 ± 306 | 240 ± 17 | 5.2 ± 1.9 | 0.03 ± 0.01 | 300 ± 111 | 1.8 ± 0.7 |
|  |  | Low-medium | 857 ± 376 | 238 ± 17 | 2.2 ± 1.0 | 0.01 ± 0.01 | 129 ± 57 | 0.8 ± 0.4 |
|  |  | Minimum-low | 1042 ± 429 | 235 ± 17 | 0.0 ± 0.0 | 0.00 ± 0.00 | 0.0 ± 0.0 | 0.0 ± 0.0 |
| Mean ± SD all respiratory classes | | | **694 ± 270** | **239 ± 2.9** | **3.4 ± 2.9** | **0.02 ± 0.02** | **198 ± 168** | **1.2 ± 1.0** |

Mean SI values, the corresponding heart position and respiratory induced cardiac movement from six controls as basis for the estimation of tidal volumes.
P(m) = P (midpoint), SD = standard deviation, SI = signal intensity.

References:

1. Magder S (2018) Heart-Lung interaction in spontaneous breathing subjects: the basics. Ann Transl Med 6:348. <https://doi.org/10.21037/atm.2018.06.19>
2. Eichinger M, Puderbach M, Smith HJ, Tetzlaff R, Kopp-Schneider A, Bock M, Biederer J, Kauczor HU (2007) Magnetic resonance-compatible-spirometry: principle, technical evaluation and application. Eur Respir J 30:972-979.

https://doi.org/10.1183/09031936.00040607

1. Röwer LM, Uelwer T, Hußmann J, Malik H, Eichinger M, Voit D, Wielpütz MO, Frahm J, Harmeling S, Klee D, Pillekamp F (2021) Spirometry-based reconstruction of real-time cardiac MRI: Motion control and quantification of heart-lung interactions. Magn Reson Med 86:2692–2702. https://doi.org/10.1002/mrm.28892
2. Koopman M, Zanen P, Kruitwagen CL, van der Ent CK, Arets, HG (2011) Reference values for paediatric pulmonary function testing: The Utrecht dataset. Respir Med *105*:15–23. https://doi.org/10.1016/j.rmed.2010.07.020
