# Supplementary material for: First experience with real-time magnetic resonance imaging-based investigation of respiratory influence on cardiac function in pediatric congenital heart disease patients with chronic right ventricular volume overload
Source: Pediatr Radiol. 2023 Oct 5;53(13):2608–21. doi: 10.1007/s00247-023-05765-9 (PMC10698081; doi:10.1007/s00247-023-05765-9)
Supplement: Supplementary file 5 — Supplementary file5 (DOCX 52.0 KB) [file 247_2023_5765_MOESM5_ESM.docx]

| RV volume | Respiratory class | | RV overload | | | | | | | | | | | |
| --- | --- | --- | --- | --- | --- | --- | --- | --- | --- | --- | --- | --- | --- | --- |
|  | Respiratory phase | Volume class | **Patient 1** | | **Patient 2** | | **Patient 3** | | **Patient 4** | | **Patient 5** | | **Patient 6** | |
|  |  |  | Estimated tidal volume (ml/cm) | RV volume | Estimated tidal volume (ml/cm) | RV volume | Estimated tidal volume (ml/cm) | RV volume | Estimated tidal volume (ml/cm) | RV volume | Estimated tidal volume (ml/cm) | RV volume | Estimated tidal volume (ml/cm) | RV volume |
| EDVi (ml/m²) | Inspiration | Minimum-low | -0.3 | 197 | -0.2 | 111 | -0.4 | 116 | 0.1 | 78 | -0.2 | 77 | -0.1 | 84 |
|  |  | Low- medium | 0.7 | 202 | 0.4 | 116 | 0.8 | 121 | 0.8 | 84 | 0.1 | 80 | 1.7 | 88 |
|  |  | Medium-high | 1.7 | 216 | 1.2 | 120 | 1.4 | 126 | 1.7 | 86 | 1.3 | 86 | 3.1 | 91 |
|  |  | High-maximum | 2.4 | 228 | 2.0 | 128 | 2.6 | 132 | 3.1 | 93 | 2.7 | 95 | 4.3 | 93 |
|  | Expiration | Minimum-low | 0.0 | 190 | 0.0 | 110 | 0.0 | 119 | 0.0 | 82 | 0.0 | 75 | 0.0 | 83 |
|  |  | Low- medium | 0.5 | 197 | 1.1 | 114 | 1.4 | 121 | 0.8 | 84 | 0.3 | 76 | 2.0 | 84 |
|  |  | Medium-high | 1.4 | 208 | 1.5 | 117 | 1.9 | 125 | 1.4 | 87 | 1.6 | 80 | 3.7 | 87 |
|  |  | High-maximum | 2.1 | 215 | 1.7 | 118 | 2.5 | 132 | 2.9 | 91 | 2.5 | 88 | 4.4 | 90 |
| ESVi (ml/m²) | Inspiration | Minimum-low | -0.3 | 64 | -0.2 | 56 | -0.4 | 50 | 0.1 | 31 | -0.2 | 27 | -0.1 | 36 |
|  |  | Low- medium | 0.7 | 67 | 0.4 | 59 | 0.8 | 52 | 0.8 | 32 | 0.1 | 27 | 1.7 | 36 |
|  |  | Medium-high | 1.7 | 73 | 1.2 | 61 | 1.4 | 53 | 1.7 | 32 | 1.3 | 28 | 3.1 | 37 |
|  |  | High-maximum | 2.4 | 77 | 2.0 | 67 | 2.6 | 55 | 3.1 | 33 | 2.7 | 30 | 4.3 | 37 |
|  | Expiration | Minimum-low | 0.0 | 60 | 0.0 | 56 | 0.0 | 50 | 0.0 | 33 | 0.0 | 25 | 0.0 | 36 |
|  |  | Low- medium | 0.5 | 63 | 1.1 | 57 | 1.4 | 51 | 0.8 | 32 | 0.3 | 25 | 2.0 | 36 |
|  |  | Medium-high | 1.4 | 69 | 1.5 | 58 | 1.9 | 51 | 1.4 | 32 | 1.6 | 26 | 3.7 | 37 |
|  |  | High-maximum | 2.1 | 71 | 1.7 | 58 | 2.5 | 53 | 2.9 | 33 | 2.5 | 28 | 4.4 | 37 |
| SVi (ml/m²) | Inspiration | Minimum-low | -0.3 | 133 | -0.2 | 55 | -0.4 | 66 | 0.1 | 47 | -0.2 | 50 | -0.1 | 48 |
|  |  | Low- medium | 0.7 | 135 | 0.4 | 57 | 0.8 | 69 | 0.8 | 52 | 0.1 | 53 | 1.7 | 52 |
|  |  | Medium-high | 1.7 | 143 | 1.2 | 59 | 1.4 | 73 | 1.7 | 54 | 1.3 | 58 | 3.1 | 54 |
|  |  | High-maximum | 2.4 | 150 | 2.0 | 67 | 2.6 | 77 | 3.1 | 60 | 2.7 | 65 | 4.3 | 56 |
|  | Expiration | Minimum-low | 0.0 | 130 | 0.0 | 54 | 0.0 | 66 | 0.0 | 49 | 0.0 | 50 | 0.0 | 47 |
|  |  | Low- medium | 0.5 | 134 | 1.1 | 57 | 1.4 | 68 | 0.8 | 52 | 0.3 | 51 | 2.0 | 48 |
|  |  | Medium-high | 1.4 | 139 | 1.5 | 59 | 1.9 | 70 | 1.4 | 54 | 1.6 | 53 | 3.7 | 50 |
|  |  | High-maximum | 2.1 | 144 | 1.7 | 59 | 2.5 | 72 | 2.9 | 57 | 2.5 | 60 | 4.4 | 53 |
| EF  (%) | Inspiration | Minimum-low | -0.3 | 68 | -0.2 | 50 | -0.4 | 57 | 0.1 | 60 | -0.2 | 65 | -0.1 | 57 |
|  |  | Low- medium | 0.7 | 67 | 0.4 | 49 | 0.8 | 57 | 0.8 | 62 | 0.1 | 66 | 1.7 | 59 |
|  |  | Medium-high | 1.7 | 66 | 1.2 | 49 | 1.4 | 58 | 1.7 | 63 | 1.3 | 67 | 3.1 | 60 |
|  |  | High-maximum | 2.4 | 66 | 2.0 | 48 | 2.6 | 58 | 3.1 | 64 | 2.7 | 68 | 4.3 | 60 |
|  | Expiration | Minimum-low | 0.0 | 68 | 0.0 | 49 | 0.0 | 57 | 0.0 | 61 | 0.0 | 67 | 0.0 | 56 |
|  |  | Low- medium | 0.5 | 68 | 1.1 | 50 | 1.4 | 57 | 0.8 | 62 | 0.3 | 67 | 2.0 | 57 |
|  |  | Medium-high | 1.4 | 67 | 1.5 | 51 | 1.9 | 58 | 1.4 | 63 | 1.6 | 67 | 3.7 | 57 |
|  |  | High-maximum | 2.1 | 67 | 1.7 | 51 | 2.5 | 58 | 2.9 | 63 | 2.5 | 68 | 4.4 | 58 |

**Supplementary Material 5: Detailed RV volumetry results**  **a**

| RV volume | Respiratory class | | Controls | | | | | | | | | | | |
| --- | --- | --- | --- | --- | --- | --- | --- | --- | --- | --- | --- | --- | --- | --- |
|  | Respiratory phase | Volume class | **Patient 7** | | **Patient 8** | | **Patient 9** | | **Patient 10** | | **Patient 11** | | **Patient 12** | |
|  |  |  | Estimated tidal volume (ml/cm) | RV volume | Estimated tidal volume (ml/cm) | RV volume | Estimated tidal volume (ml/cm) | RV volume | Estimated tidal volume (ml/cm) | RV volume | Estimated tidal volume (ml/cm) | RV volume | Estimated tidal volume (ml/cm) | RV volume |
| EDVi (ml/m²) | Inspiration | Minimum-low | -0.1 | 63 | 0.1 | 63 | -0.1 | 86 | 0.1 | 72 | -0.1 | 65 | -0.1 | 82 |
|  |  | Low- medium | 0.3 | 66 | 1.1 | 74 | 0.1 | 90 | 0.4 | 75 | 0.3 | 70 | 0.1 | 84 |
|  |  | Medium-high | 1.6 | 71 | 2.2 | 79 | 1.0 | 93 | 1.1 | 81 | 0.9 | 72 | 1.8 | 90 |
|  |  | High-maximum | 3.2 | 77 | 3.6 | 92 | 2.9 | 99 | 1.9 | 85 | 1.6 | 76 | 2.7 | 92 |
|  | Expiration | Minimum-low | 0.0 | 60 | 0.0 | 59 | 0.0 | 87 | 0.0 | 74 | 0.0 | 65 | 0.0 | 81 |
|  |  | Low- medium | 0.8 | 62 | 1.5 | 64 | 0.7 | 89 | 0.5 | 76 | 0.5 | 66 | 0.6 | 82 |
|  |  | Medium-high | 2.0 | 66 | 3.2 | 66 | 1.9 | 91 | 1.1 | 77 | 1.1 | 70 | 1.7 | 85 |
|  |  | High-maximum | 3.0 | 71 | 4.0 | 78 | 2.9 | 95 | 1.7 | 80 | 1.6 | 73 | 2.7 | 88 |
| ESVi (ml/m²) | Inspiration | Minimum-low | -0.1 | 25 | 0.1 | 24 | -0.1 | 42 | 0.1 | 29 | -0.1 | 30 | -0.1 | 36 |
|  |  | Low- medium | 0.3 | 26 | 1.1 | 26 | 0.1 | 42 | 0.4 | 29 | 0.3 | 30 | 0.1 | 36 |
|  |  | Medium-high | 1.6 | 26 | 2.2 | 27 | 1.0 | 42 | 1.1 | 30 | 0.9 | 31 | 1.8 | 36 |
|  |  | High-maximum | 3.2 | 26 | 3.6 | 28 | 2.9 | 43 | 1.9 | 30 | 1.6 | 31 | 2.7 | 37 |
|  | Expiration | Minimum-low | 0.0 | 24 | 0.0 | 23 | 0.0 | 41 | 0.0 | 28 | 0.0 | 29 | 0.0 | 35 |
|  |  | Low- medium | 0.8 | 24 | 1.5 | 25 | 0.7 | 41 | 0.5 | 28 | 0.5 | 29 | 0.6 | 35 |
|  |  | Medium-high | 2.0 | 24 | 3.2 | 24 | 1.9 | 42 | 1.1 | 29 | 1.1 | 30 | 1.7 | 35 |
|  |  | High-maximum | 3.0 | 25 | 4.0 | 26 | 2.9 | 42 | 1.7 | 29 | 1.6 | 30 | 2.7 | 36 |
| SVi (ml/m²) | Inspiration | Minimum-low | -0.1 | 38 | 0.1 | 38 | -0.1 | 44 | 0.1 | 43 | -0.1 | 36 | -0.1 | 46 |
|  |  | Low- medium | 0.3 | 40 | 1.1 | 48 | 0.1 | 49 | 0.4 | 46 | 0.3 | 40 | 0.1 | 48 |
|  |  | Medium-high | 1.6 | 45 | 2.2 | 53 | 1.0 | 51 | 1.1 | 51 | 0.9 | 42 | 1.8 | 54 |
|  |  | High-maximum | 3.2 | 51 | 3.6 | 64 | 2.9 | 56 | 1.9 | 55 | 1.6 | 46 | 2.7 | 55 |
|  | Expiration | Minimum-low | 0.0 | 36 | 0.0 | 36 | 0.0 | 45 | 0.0 | 45 | 0.0 | 36 | 0.0 | 45 |
|  |  | Low- medium | 0.8 | 38 | 1.5 | 40 | 0.7 | 47 | 0.5 | 47 | 0.5 | 37 | 0.6 | 47 |
|  |  | Medium-high | 2.0 | 42 | 3.2 | 42 | 1.9 | 49 | 1.1 | 49 | 1.1 | 40 | 1.7 | 49 |
|  |  | High-maximum | 3.0 | 47 | 4.0 | 52 | 2.9 | 53 | 1.7 | 51 | 1.6 | 43 | 2.7 | 53 |
| EF  (%) | Inspiration | Minimum-low | -0.1 | 60 | 0.1 | 61 | -0.1 | 52 | 0.1 | 59 | -0.1 | 54 | -0.1 | 56 |
|  |  | Low- medium | 0.3 | 61 | 1.1 | 65 | 0.1 | 54 | 0.4 | 62 | 0.3 | 57 | 0.1 | 57 |
|  |  | Medium-high | 1.6 | 63 | 2.2 | 66 | 1.0 | 55 | 1.1 | 63 | 0.9 | 58 | 1.8 | 60 |
|  |  | High-maximum | 3.2 | 66 | 3.6 | 70 | 2.9 | 57 | 1.9 | 65 | 1.6 | 60 | 2.7 | 60 |
|  | Expiration | Minimum-low | 0.0 | 61 | 0.0 | 61 | 0.0 | 52 | 0.0 | 61 | 0.0 | 55 | 0.0 | 56 |
|  |  | Low- medium | 0.8 | 62 | 1.5 | 62 | 0.7 | 53 | 0.5 | 63 | 0.5 | 55 | 0.6 | 57 |
|  |  | Medium-high | 2.0 | 63 | 3.2 | 63 | 1.9 | 54 | 1.1 | 63 | 1.1 | 57 | 1.7 | 58 |
|  |  | High-maximum | 3.0 | 65 | 4.0 | 67 | 2.9 | 56 | 1.7 | 64 | 1.6 | 59 | 2.7 | 60 |

**b**

Detailed presentation of the RV volumetry results of **a** RV overload patients and **b** controls.

*EDVi* end-diastolic volume indexed to body surface area, *EF* ejection fraction, *ESVi* end-systolic volume indexed to body surface area, *RV* right ventricular, *SVi* stroke volume indexed to body surface area.
